# Modifying Membranotropic Action of Antimicrobial Peptide Gramicidin S by Star-like Polyacrylamide and Lipid Composition of Nanocontainers

**DOI:** 10.3390/ijms25168691

**Published:** 2024-08-09

**Authors:** Olga V. Vashchenko, Volodymyr P. Berest, Liliia V. Sviechnikova, Nataliya V. Kutsevol, Natalia A. Kasian, Dmitry S. Sofronov, Oleksii Skorokhod

**Affiliations:** 1 Institute for Scintillation Materials of NAS of Ukraine, 60 Nauky Ave., 61172 Kharkiv, Ukraine; olga_v@isma.kharkov.ua (O.V.V.); l.budjanskaja92@gmail.com (L.V.S.);; 2 Department of Molecular and Medical Biophysics, V. N. Karazin Kharkiv National University, 4 Svobody Sq., 61022 Kharkiv, Ukraine; 3 Research Department, Taras Shevchenko National University of Kyiv, 60 Volodymyrska St., 01601 Kyiv, Ukraine; kutsevol@ukr.net; 4 State Scientific Institution “Institute for Single Crystals” of NAS of Ukraine, 60 Nauky Ave., 61072 Kharkiv, Ukraine; 5 Department of Life Sciences and Systems Biology, University of Turin, Via Accademia Albertina 13, 10123 Turin, Italy

**Keywords:** gramicidin S, antimicrobial peptide, drug delivery, nanocontainers, differential scanning calorimetry DSC, Fourier transform infrared spectroscopy FTIR, lipid membranes

## Abstract

Gramicidin S (GS), one of the first discovered antimicrobial peptides, still shows strong antibiotic activity after decades of clinical use, with no evidence of resistance. The relatively high hemolytic activity and narrow therapeutic window of GS limit its use in topical applications. Encapsulation and targeted delivery may be the way to develop the internal administration of this drug. The lipid composition of membranes and non-covalent interactions affect GS’s affinity for and partitioning into lipid bilayers as monomers or oligomers, which are crucial for GS activity. Using both differential scanning calorimetry (DSC) and FTIR methods, the impact of GS on dipalmitoylphosphatidylcholine (DPPC) membranes was tested. Additionally, the combined effect of GS and cholesterol on membrane characteristics was observed; while dipalmitoylphosphatydylglycerol (DPPG) and cerebrosides did not affect GS binding to DPPC membranes, cholesterol significantly altered the membrane, with 30% mol concentration being most effective in enhancing GS binding. The effect of star-like dextran-polyacrylamide D-g-PAA(PE) on GS binding to the membrane was tested, revealing that it interacted with GS in the membrane and significantly increased the proportion of GS oligomers. Instead, calcium ions affected GS binding to the membrane differently, with independent binding of calcium and GS and no interaction between them. This study shows how GS interactions with lipid membranes can be effectively modulated, potentially leading to new formulations for internal GS administration. Modified liposomes or polymer nanocarriers for targeted GS delivery could be used to treat protein misfolding disorders and inflammatory conditions associated with free-radical processes in cell membranes.

## 1. Introduction

Decapeptide gramicidin S (GS) is a widely used effective antimicrobial drug. Its pharmacological action is commonly associated with direct binding to membranes of bacterial and eukaryotic cells, which provide both therapeutically positive antibacterial effects and negative hemolytic ones [1,2,3,4,5,6,7,8,9,10,11,12,13].

The primary structure of GS is known: it is a cyclic, C2-symmetrical decapeptide with the sequence cyclo(Pro-DPhe-Leu-Orn-Val)2 [6]. The synthesis of gramicidin S is a strictly regulated process that involves precise peptide chemistry and enzyme specificity. The enzymatic formation of GS from its precursor peptides is very similar to enzyme-catalyzed protein post-translational modifications. Conformation studies solve the secondary structure of GS as an anti-parallel β-sheet with four intermolecular hydrogen bonds [14], which generally maintains both in water and in membrane surroundings.

Nowadays, a broad range of features regarding the biological activity of GS has been identified; however, the detailed molecular mechanism of GS action is still far from clear. Anyway, the primary GS targets are lipid membranes. GS partitioning into lipid membranes is predominantly driven by large favorable entropy change [9]. After being introduced into the membrane interior, GS molecules bonded directly underneath the polar lipid part, pointing its charged residues toward the water phase [3,5,13]. GS is characterized by two types of binding to lipid membranes, namely monomeric and oligomeric [2,10]. GS oligomers are mainly observed at GS concentration above 2 mol %, and it is worth noting that the bactericidal effect of GS is observed at the same concentration [11].

It was assumed that GS binding to model lipid membranes results in pore formation and, therefore, cell lysis [15]. However, novel data reveal that over the pore formation, GS-induced large lipid domain formation coupled with changes of localization of peripheral proteins take place in vivo in bacterial cells [16]. It has also been established that GS introduction in artificial lipid membranes causes elevation of lipid cross-area, as well as diminution in lipid chains ordering, hydrophobic thickness, and interlamellar distance of the membranes [5]. Taken together, these findings point to the disordering and dehydrating membranotropic effects of GS, which need to be further studied.

Differences in membrane lipid compositions among various biological species, especially between eukaryotes and prokaryotes, suggest a method for differentiating the therapeutic actions of membranoactive drugs like GS while minimizing their negative side effects, such as hemolysis, in animal and human cells [6,17]. Indeed, lipid composition and asymmetry are able to predetermine membrane permeability for GS [18,19] and, therefore, could impact drug–membrane interactions. Indeed, changes in lipid composition are pointed out as one of the mechanisms of cell resistance to antibiotics [20,21]. This is in line with the dependence of the GS effect on lipid composition, which has been reported, especially on membrane hydrophilic parts and on the presence of cholesterol (Chol) [1,8,9,10,22,23]. Interestingly, one study [14] reports that a Chol content of about 40% prevents GS from binding to the membrane, while 10% Chol content favors this binding. Thus, the Chol effect on GS–membrane interaction needs to be further studied.

The importance of in-depth studies on GS also stems from the fact that GS, among other antimicrobial peptides, attracts the attention of researchers addressing the pressing issue of widespread microbial resistance to conventional antibiotics. This is due to its non-specific mechanism of action and the much slower development of resistant microbial strains.

It is also important to note that in the last decades, a number of GS analogs were synthesized with different lengths of the peptide chain and various numbers of charged groups, with the introduction of post-translational modifications and different positioning of hydrophobic amino acids in the moiety. Some of them have essential preferences over the original GS in a therapeutic index. Nevertheless, it would take additional years of study and clinical trials to introduce GS analogs as a new drug into clinical practice. Our idea is to explore the possibilities of modulation of “standard” GS binding effects by means of its application in tandem with other certified pharmaceuticals, as well as by using modern nanocarriers for targeted delivery. Assuming the possibility of applying lipid nanocarriers (liposomes and micelles) or polymer nanocarriers for GS encapsulation and delivery, we have studied here the impact of lipid composition (particularly, cholesterol content) and calcium ions on GS interaction with these drug-delivery vehicles.

Taking into account that the principal distinguishing feature of lipid composition of eukaryotic cells comparatively from bacterial ones is the presence of phosphatidylcholines and Chol, several types of model lipid membranes were investigated here: (1) pure dipalmitoylphosphatidylcholine (DPPC) membrane, which is widely used as model lipid membrane [24]; (2) membrane consisting of DPPC and dipalmitoylphosphatidylglycerol (DPPC-DPPG membrane), which mimics negative membrane charge of Gram-positive bacteria [25,26]; (3) membrane containing DPPC and cerebrosides (DPPC-Cer membrane), which mimics membranes of skin cells as well as some bacterial cells [26,27]; (4) cholesterol-containing membrane (DPPC-Chol membrane), which mimics membranes of mammalian cells.

Another way to modulate GS binding and, thus, its biological effects could be achieved through various guest substances. For example, it has been shown that the formation of an intermolecular complex between GS and the lipopeptide surfactin can lead to a rare case of bacterial resistance to GS [28]. As another example, the modulation of GS is reported under Ca^2+^ introduction in concentrations above 100 mM [11]. In this study, we will also test the Ca^2+^ effect on GS binding to the complex membranes.

A powerful way to modulate drug–host interactions and drug efficacy is by using polymers (polymeric drug delivery systems). For instance, star-like dextran-polyacrylamide copolymers have been previously examined as drug carrier nanocontainers [29,30,31,32] and as membranotropic agents [33]. Based on the published results, the polyanionic form D-g-PAA(PE) of dextran-graft-polyacrylamide copolymer D-g-PAA is supposed as a perspective modulator of GS action and is studied here.

Finally, some non-canonical applications of GS are reported in the literature: GS anti-aggregatory activity towards blood clot system [34], as well as activity against formation of protein fibrillar aggregates [35] and radioprotective properties [36]. Elucidation of governing molecular mechanisms of GS membranotropic effects [37] would accelerate the development of new effective formulations for treating protein (mis)folding deceases and inflammatory disorders associated with free-radical processes in cell membranes.

The aim of the present study is to explore various factors affecting GS–membrane interactions, such as membrane cholesterol content, the presence of star-like polyanionic dextran-polyacrylamide copolymer D-g-PAA(PE), and calcium ions in order to expand the possibilities for optimizing the therapeutic action of GS. The modulation of GS membranotropic effects, observed here, contributes to exploring new ways to redeploy this certified and successful pharmaceutical.

## 2. Results

### 2.1. Impact of Cholesterol Content

**Figure 1:**
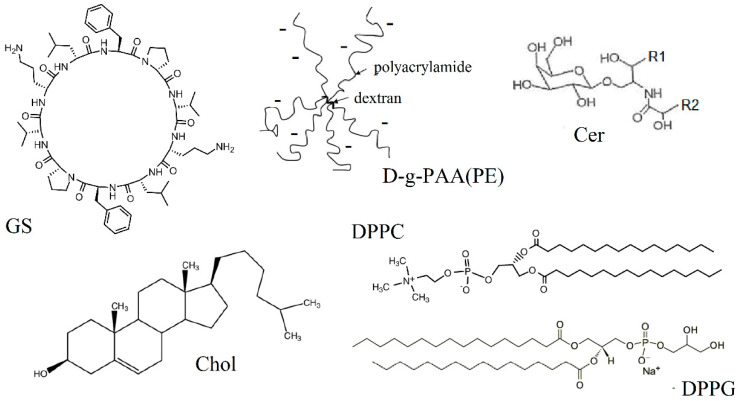
Chemical structures of decapeptide gramicidin S (GS), dipalmitoylphosphatidylcholine (DPPC), dipalmitoylphosphatydylglycerol (DPPG), cholesterol (Chol), and cerebroside (Cer, R1, and R2 are fatty acid residues) and schematic representation of star-like polyanionic dextran-polyacrylamide copolymer D-g-PAA(PE).

The chemical structure of GS and the main GS-interacting molecules studied here are presented in Figure 1. We applied DSC to study the impact of cholesterol on GS binding with the DPPC membrane. Firstly, we tested the effect of pure GS on the DPPC membrane and distinguished GS oligomers and polymers. Then, we tested four types of membranes for GS binding, three control membranes, and membrane with cholesterol content: (1) dipalmitoylphosphatidylcholine (DPPC) membrane; (2) membrane consisting of DPPC and dipalmitoylphosphatidylglycerol (DPPC-DPPG membrane); (3) membrane containing DPPC and cerebrosides (DPPC-Cer membrane); (4) cholesterol-containing membrane (DPPC-Chol membrane).

**Figure 2:**
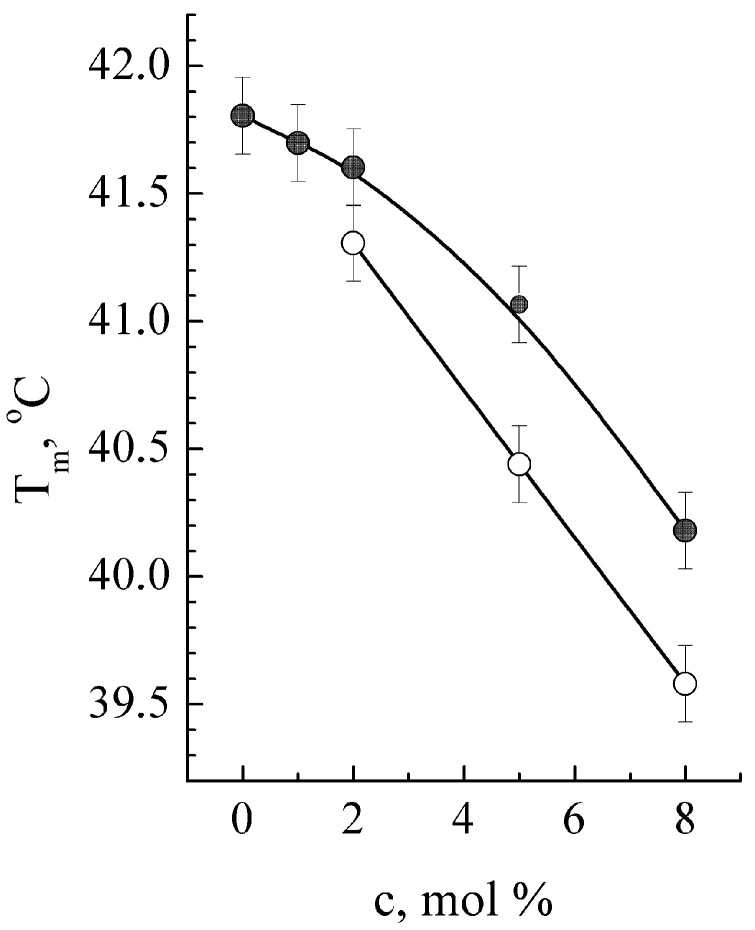
Dependences of melting temperature of DPPC (T_m_) on GS concentration (c, mol %). Solid circles are plotted for the initial DSC peak and GS monomer binding; data attributed to the binding of GS oligomers are marked with open circles. Means ± SDs for 3–5 independent preparations are shown.

The original DSC thermogram of neat DPPC membrane contains two endothermal peaks: the low-temperature peak is near 36 °C and corresponds to the phase transition “gel to ripple phase” called pre-transition (T_p_). Another peak reflects the “gel to liquid crystal” phase transition called membrane melting (T_m_). These parameters were used to distinguish the GS binding under monomer and oligomer forms and study the dose dependence of DPPC membrane T_m_ from GS (Figure 2).

DSC data evidence that all studied types of membranes are impacted by GS in a similar way and appeared in thermograms as a lowering of T_m_ (Figure 2, Table 1) and in the disappearance of the pre-transition T_p_ peak. The most pronounced T_m_ shift (ΔT_m_) and half-width widening of the melting peak ΔT_m½_ is observed for the DPPC-Chol membrane (Table 1). Importantly, T_m_ shift in the membrane containing both Chol and GS is discernibly non-additive to the one containing individual components. Indeed, in the membranes containing only GS at 5 mol %, the temperature shift ΔT_m_ = −1.5 ± 0.15 °C (Figure 2) was observed; Chol individually (10 mol %) induced ΔT_m_ = −1.7 ± 0.15 °C, whereas being introduced together, GS and Chol led to ΔT_m_ = −5.3 ± 0.15 °C.

In addition, GS introduction into the DPPC membrane at a concentration above 2 mol % causes a substantial increase in the asymmetry of DSC profiles, indicating the appearance of a new low-temperature DSC peak, which could be specified as T_m_*. According to the literature data on two possible modes of GS binding to lipid membranes (in the form of GS monomers or oligomers) [10], the T_m_* peak is attributed here to GS oligomer binding. The dependence of T_m_ on GS concentration is shown in Figure 2 for both possible modes of GS binding—monomeric and oligomeric. The additionally observed increase in the half-width (the width of a spectral peak at half of its maximum amplitude) of the “oligomeric” DSC peak at higher GS concentration suggests that the lipids in the membrane become much less homogeneous in the presence of GS.

A comparison of membranotropic effects of GS was performed using four different DPPC-based membranes with GS content of 5 mol %. Table 1 presents the most sensitive thermodynamic parameters of membrane melting, such as molar membranotropic activity a*_mol_* [38], defined as T_m_ shift per unit concentration, half-width of the melting peak ΔT_m1/2_, and shift of the melting enthalpy ΔΔ*H_m1/2_*. GS induces a low-temperature shift of membrane melting peak in all tested types of membranes, with the most pronounced effect in the DPPC-Chol membrane (Table 1, row 4). This result indicates an increased distribution of GS into the DPPC-Chol membrane, demonstrating the synergistic effect of GS and Chol towards altering the DPPC membrane and is in sync with the general Chol effect on biological membranes [39,40,41].

To further investigate the observed synergistic effect between GS and Chol in the DPPC membrane, two additional steps were performed. Firstly, all original DSC profiles were decomposed into two components, which correspond, in accordance with [40], to Chol-depleted (peak T_m_) and Chol-enriched (peak T_m_*) lipid phases. High values of determination coefficient (R^2^ > 0.99) validate the correctness of the decomposition procedure.

**Figure 3:**
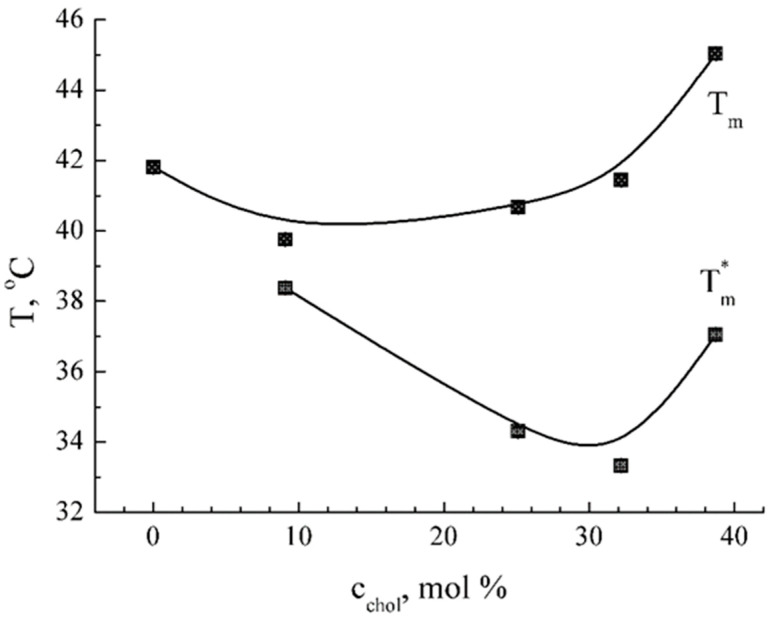
Effect of Chol content on thermodynamic parameters of GS binding with the DPPC membrane. The peak temperatures of Chol-enriched (lower four points with correspondent approximate curve, marked T_m_*) and Chol-depleted lipid phases (upper five points with correspondent approximate curve, marked T_m_) are plotted against Chol concentration in the DPPC membrane containing GS at 5 mol %. Data from one representative experiment out of three independent preparations.

Secondly, Chol content (c_chol_) has been varied in our experimental model membrane systems in the range of 0 to 40 mol %. As shown in Figure 3, the dependence T_m_* from c_chol_ is non-linear, with a clear minimum at Chol content of about 30% *w*/*w*. The dependence T_m_ from c_chol_ has a similar non-linear trend with the minimum at 10–30 mol %. The same critical value c_chol_ of approximately 30 mol % is found on concentration dependences of the peaks’ half-width, whereas enthalpy values decrease monotonously with Chol concentration.

### 2.2. Impact of Lipid Composition

FTIR spectroscopy has been applied to provide important information on the impact of lipid composition on GS–membrane interactions. Note that the control DPPC membrane FTIR spectra contained mainly the sets of characteristic bands of DPPC and water, as they were identified according to the literature data [42,43,44,45,46]. The characteristic bands of GS, Chol, and lipid components were detected separately on FTIR spectra of corresponding pure substances. The bands were modified or absent when the spectra of the complex systems were examined. The possible impact of single compounds’ bands has been taken into account during FTIR spectra analysis.

GS introduction into the DPPC-DPPG membrane caused changes in relative intensity between two components of the vibration band of DPPC phosphate groups (νP=O) at 1220 cm^−1^ and 1240 cm^−1^, showing the difference from the DPPC membrane. According to previously described FTIR spectra analysis and interpretation [46], these components correspond to asymmetric valence vibrations of hydrated and “free” phosphate groups, respectively. They are considered extremely sensitive markers of structural rearrangements in the membrane water shell, particularly during interactions with exogenous substances [41,47]. An increase in relative intensity of the 1220 cm^−1^ band in the DPPC-DPPG membrane containing GS indicates dehydration of the membrane surface or GS binding to phosphate groups of lipids, as was previously reported [48]. Note that this effect could not be a result of band superposition because the nearest bands of DPPG at 1150 cm^−1^ and 1260 cm^−1^ did not change. The effect of dehydration of membrane lipid P=O groups by introducing Chol alone appeared to be similar to that of GS, but under the simultaneous introduction of Chol and GS into the DPPC membrane, opposite compensative effects were observed.

Vibration bands of methyl groups were characterized using peak decomposition onto four components (for stretching vibrations νCH_2_ 2920 cm^−1^ and 2850 cm^−1^) or onto three components (for bending vibrations δCH_2_ 1470 cm^−1^). The values of peak maximum and half-width were defined for each of these components. High values of the determination coefficient (*R*^2^ > 0.99) validate the correctness of the decomposition procedure. The obtained results evidence that Chol introduction causes the narrowing of νCH_2_ bands coupled with their bathochromic shift, which reflects increasing both lipid homogeneity and fluidity. GS addition enhanced this effect in accordance with the GS-Chol synergic effect observed here by the DSC method (Table 1).

Analysis of characteristic bands of carbonyl groups of DPPC νC=O 1734 cm^−1^ was performed according to the established procedure [49,50]. The original νC=O bands were decomposed into two constituent peaks (low-frequency and high-frequency), which correspond to hydrated (C=O_hydr_) and non-hydrated (C=O_free_) carbonyl groups. According to published data [49,50], the ratio of corresponding peak areas (C=O_hydr_/C=O_free_) reflects the portion of hydrated carbonyl groups and, therefore, allows monitoring of changes of water access into the membrane interface. This value amounts to 2.1 for neat DPPC membrane, 2.0 for DPPC-Chol membrane, and 3.5 for DPPC-Chol membrane containing 8 mol% GS. Both Chol and GS cause hypsochromic shifts of νC=O bands.

FTIR data for a set of multi-compound lipid membranes containing GS show that GS has qualitatively and quantitatively different impacts depending on lipid composition (Table 2). In negatively charged DPPC-DPPG membrane, GS causes dehydration of phosphate groups coupled with disordering of alkyl chains of lipids, as concluded from ν_as_CH_2_ and δCH_2_ increase (Table 2, first line), whereas an opposite effect is observed in DPPC-Chol membrane (Table 2, third line). Note that in multi-compound membranes, there is no clear correlation between alkyl chain ordering and hydration of membrane surface [51]. Present data contribute to clear this issue.

### 2.3. Impact of a Polymer Carrier

The dextran-graft-polyacrylamide copolymer in its anionic form D-g-PAA(PE) was chosen as a potential modulator of GS binding with membrane. According to [31,52], D-g-PAA(PE) molecular weight is about 7 kDa, and the hydrodynamic radius is approximately 70 nm. It contains five polyacrylamide chains with approximately 37% negatively charged group moiety (Figure 1). In our previous studies, we examined the potential of D-g-PAA(PE) to be used as a drug carrier [29] and its ability to bind to a model lipid membrane [33].

**Figure 4:**
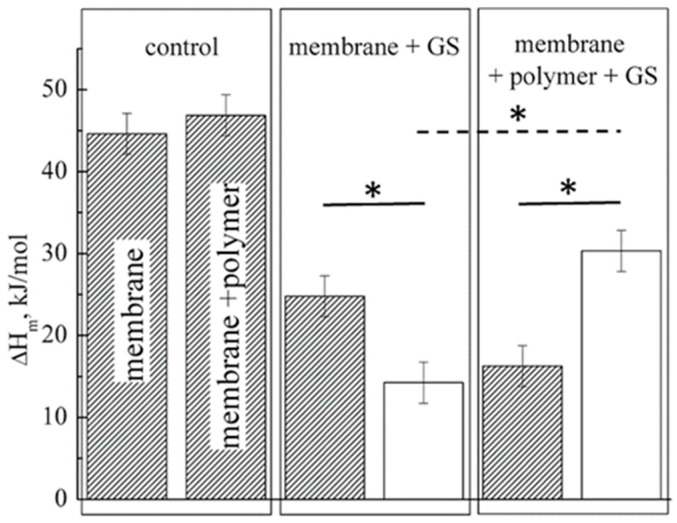
Values of enthalpies of DSC peaks of DPPC membrane (showed as “membrane”) in the presence of GS and/or polymer D-g-PAA(PE) (showed as “polymer”). Empty columns correspond to the enthalpies of GS “oligomer” peaks. Data are shown for GS content 5 mol %. Means ± SDs of 3–5 independent preparations are shown. Significant changes with *p* < 0.05 are indicated with * over solid lines for the effect of GS oligomer binding and over dotted line for the effect of D-g-PAA(PE) polymer.

In this study, the impact of D-g-PAA(PE) on GS binding with membranes was initially observed at a relatively high GS concentration (about 8 mol %) and manifested itself as an increase in T_m_, T_m_*, and T_m*½_ in DCS thermograms of DPPC membranes. These changes in lipid transition temperatures appeared exactly the opposite of what we observed for the binding of free GS to membranes (Figure 2). This indicates that D-g-PAA(PE) prevents GS binding with DPPC membrane at high GS concentrations. At the same time, at a moderate GS concentration of 5 mol %, the presence of D-g-PAA(PE) polymer changes the affinity of GS binding with membranes, favoring the incorporation of peptide oligomers into lipid bilayers versus monomeric forms of GS. This could be figured out from the redistribution of the DSC peak enthalpies between membranes with GS bound in a monomeric form and GS bound as oligomers (Figure 4, empty columns for GS oligomers). Enthalpy ΔH_m_ was significantly (2.1 ± 0.3 times) higher for GS bounds in oligomeric forms due to polymer D-g-PAA(PE) presence (Figure 4). This result means the facilitation of GS oligomeric binding mode at the expense of monomeric form.

### 2.4. Impact of Calcium Ions

**Figure 5:**
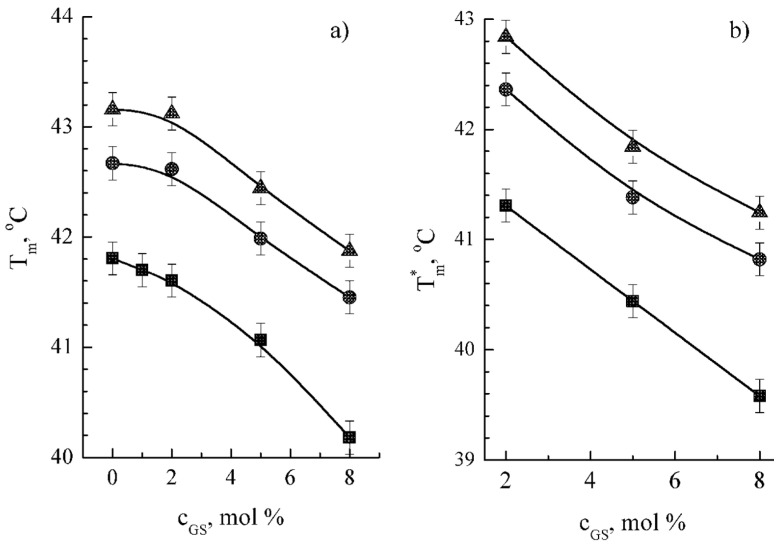
Dependences of T_m_ (panel **a**) and T_m_* (panel **b**) on GS content in DPPC membranes prepared on water subphase (■), 100 мM CaCl_2_ (●), and 200 мM CaCl_2_ (▲).

The importance of calcium ions as moderators of membrane interactions with exogenic substances is well-known [11,53,54], but further studies are needed. In order to clarify the impact of calcium ions on GS–membrane interactions, DSC thermograms were obtained for DPPC membrane containing GS at different concentrations of CaCl_2_ (Figure 5). We observed the positive shifts of phase transition temperatures after the increase in ionic strength (Figure 5). These data are in accordance with the individual membranotropic effect of Ca^2+^ ions [55], whereas the effect of Cl^−^ ions is known to be neglectable [56]. However, the shape of the dependence curves T_m_ from GS concentration remains similar for different Ca^2+^ concentrations (Figure 5). The equidistance of the dependence curves was interpreted as the additivity of GS and CaCl_2_ effects in the DPPC membrane. Enthalpies calculated from DSC thermograms were changed insignificantly. Taking into account opposite directions of GS versus CaCl_2_ membranotropic effects, significant peak broadening observed in DSC thermograms from the systems containing CaCl_2_ is plausibly caused by independent binding of GS and Ca^2+^ with the membranes.

## 3. Discussion

GS binding to biological membranes is a crucial step in its activity. Two modes, monomeric and oligomeric, of GS binding to model lipid membranes were established here using calorimetry methods by observing an additional low-temperature peak of DPPC membrane melting. DSC profiles of the melting suggest simultaneous binding of GS monomers and oligomers to model biological membranes.

Possibilities of modulation of GS–membrane interactions were explored in this study using various factors, such as Chol content, lipid composition, calcium ions, and a polymer D-g-PAA(PE) carrier.

Critical Chol content was monitored and established here as 30 mol % (Figure 3) by DSC. It was concluded from the analysis of the qualitative change of GS binding to membrane, from facilitating to hindering. Additionally, the ability of lipid membrane composition to substantially modify GS membranotropic effect was shown using FTIR data (Table 2). We show that Chol’s impact on GS binding was much stronger than the effect of DPPG or ceramides. This effect can be interpreted as synergism and is in accordance with the literature data on ^19^F NMR [10], which demonstrated enhancement of GS binding to membranes in the presence of similar concentrations of Chol. The specific effect of Chol in phospholipid membranes has been reported before [39] when the enhancement of spectrin distribution into lipid membranes in the presence of Chol was observed. This effect was explained by increasing phase boundaries, which serve as binding sites of spectrin in spectrin-containing membranes. The same mechanism could be proposed for GS in the presence of Chol. Indeed, according to [40,41], Chol content of about 10% *w*/*w* causes non-homogeneity of lateral lipid ordering and an increase in membrane-free volume at the region of the membrane interface, i.e., in the area of GS localization in the membrane. In our opinion, the totality of these features indicates significant differences in DPPC membrane properties when the Chol content is much below or above 30 mol %. The composition with low Chol (10–30 mol %) promotes GS binding to the lipid bilayer, whereas 40 mol % hinders the binding. It is noteworthy that exactly the same 30 mol % composition corresponds to a homogenous DPPC-Chol membrane in a single liquid-ordered phase described by others [40]. These findings integrate with previous data obtained by NMR analysis [10,41] concerning the opposite Chol effect at concentrations of 10 mol % and 40 mol %. Taking into account the absence of sterols in bacterial cells and the presence in eukaryotes, our finding could be applied to explain the harmful side effects of GS on human cells, e.g., erythrocytes hemolysis [6]. In summary, the cholesterol concentration of 30 mol % is found to maximally influence DSC and FTIR parameters, meaning the most effective GS binding to membranes. These data are in accordance with the known Chol effect of blurring phase transition peaks in lipid membranes [40].

FTIR analysis, performed here, is based on established protocols [42,43,44,45,46,47,48,49,50], and the obtained data correspond with similar membrane systems tested for drugs. It is important to note that, generally, in multi-compound membranes, there is no clear correlation between alkyl chain ordering and membrane surface hydration [51]. Our data contribute to clearing this issue.

Generally, it was shown that the composition of lipid membranes can modify the activity of antibacterial peptides [10], which is very important for pharmacology. In concordance with results obtained by other authors [16], our data contribute to the understanding of the changes in lipids’ localization in membrane and lipid domain formation caused by the presence of GS.

The data on the interactions of GS with D-g-PAA(PE) inside the membrane indicate that D-g-PAA(PE) partially prevents GS from binding to the DPPC membrane. In general, these interactions could be direct or membrane-mediated. The direct type of interaction is probably realized by the formation of intermolecular complexes between the cationic drug (GS) and the polyanionic polymer D-g-PAA(PE). However, this process is hampered by the majority of ions in the HBSS medium, which are able to create shells of counter-ions around both GS and D-g-PAA(PE). The second membrane-mediated type of interaction could consist of the concurrent binding of both substances with the experimental membranes. Naturally, both mechanisms could simultaneously take place. In summary, it was observed that a polymer carrier D-g-PAA(PE) strongly modulates GS interaction with the lipid membrane by diminishing its total membranotropic effect and favoring the binding of GS oligomers at the expense of monomeric binding (Figure 4). Thus, D-g-PAA(PE) could be preliminarily proposed as a perspective delivery system for GS, which needs to be additionally studied.

Alongside numerous important functions in living organisms, calcium ions serve as moderators of membrane interactions with various exogenic substances [11,53,54]. Independent binding of GS and calcium ions to the membrane was observed here under calcium concentrations up to 200 mM (Figure 5). This could be an additional regulatory factor for GS binding to nanocarriers or for the GS mechanism of biological action, including GS-induced hemolysis.

The obtained results on GS interaction with lipid components permit the proposal of GS for the creation of optimized or new drug formulations, as well as specially formulated liposomes or polymer nanocarriers, which could be used for targeted internal delivery of GS for such important diseases as protein misfolding diseases and inflammatory disorders associated with free-radical processes in cell membranes.

## 4. Materials and Methods

Gramicidin S (GS, PubChem CID 73357) was used as chemically pure gramicidin S from Bacillus brevis (“Sigma-Aldrich, Merk”, St. Louis, MO, USA, 50847, 90% pure by HPLC) or in some control tests as pharmaceutical preparation after additional purification. No difference was observed by applying the substance from these two sources. Chemically pure *L*-α-dipalmitoylphosphatidylcholine (DPPC), *L*-α-dipalmitoylphosphatidylglycerol sodium salt (DPPG), and cholesterol (Chol) were purchased from “Sigma-Aldrich”. Cerebrosides (Cer) were purchased from “BASF” (Ceramides LS 3773). Star-like polyanionic dextran-polyacrylamide copolymer D-g-PAA(PE) (Figure 1) was synthesized by one of the co-authors, Dr. N.V. Kutsevol, in Taras Shevchenko National University of Kyiv, Ukraine. Commercially available ultra-pure CaCl_2_ solution “calcium chloride” was used (Darnitsa Pharmaceutical Company, Kyiv, Ukraine).

### 4.1. Membrane Preparation

Lipid DPPC membranes were prepared according to the following procedure. Lipid compounds for (i) basal DPPC membranes, (ii) control membranes DPPC-DPPG and DPPC-Cer (see below), and (iii) Chol dose-dependence experiments and GS were dissolved in chemically pure ethanol. The GS quantity was adjusted to provide GS content in membrane systems from 1 to 8 mol %. The solvent was then evaporated by means of Concentrator Plus equipment (“Eppendorf”, Hamburg, Germany) for 6 to 10 hours at 45 °C and 0.2 mbar. After drying, the samples were hydrated with Hanks’ balanced salt solution without phenol red (HBSS) at pH 7.4 (“Thermo Fisher Scientific”, Waltham, MA, USA) to provide a water content of 60% *w*/*w*. The membrane samples were used immediately after the preparation procedure (if needed, they were stored at 5 °C, with recurrent heating up to 50 °C, combined with careful mixing and quality control). Generally, the lipid structures obtained this way are stable for at least 10 days; we performed DSC analyses, which showed that the parameters of the membrane varied only within experimental errors during this period. The accuracy of drying, hydration, and incubation stages was controlled by the samples’ precise weight using micro-balance Mettler XP26 (“Mettler-Toledo”, Columbus, OH, USA).

The compositions of control membranes were DPPC:DPPG 50:50 *w*/*w* and DPPC:Cer 90:10 *w*/*w*. In DPPC-Chol membranes, the ratio DPPC:Chol was from 92:8 to 60:40 mol %. Water-soluble components were introduced into the experimental system in the form of solution at the hydration stage: D-g-PAA(PE) at 2% *w*/*w* in HBSS and 10% *w*/*w* solution of calcium chloride. Unless otherwise indicated, the experiments were conducted at room temperature (approximately 21 °C).

The type of obtained membranes are large multi-bilayer vesicles, i.e., stacks of planar lipid bilayers [19]. These lipid structures, with a curvature radius much larger than a bilayer thickness, correspond well to cytoplasmic cell membranes, which could be considered planar. Similar planar lipid films have also been used to study several AMPs [57,58]. The value of ζ-potential could not be accurately measured for our DPPC membranes, which consist of multilamellar vesicles containing 40 % *w/w* lipids. To better understand the ζ-potential, we analyzed DPPC monolamellar liposomes with a lipid content of 0.025 % *w/w*. For these liposomes, we detected a ζ-potential value ranging from −5 to −9 mV at pH 7.4 in Hanks’ balanced salt solution without phenol red. The mean hydrodynamic diameter of the liposomes was estimated to be 0.5 nm, with a polydispersity index of approximately 0.37. Nevertheless, given the large variability in the data obtained, we conclude that this method is unlikely to be useful in revealing the impact of lipid composition in our experimental system.

### 4.2. DSC Investigations

The differential scanning calorimetry (DSC) technique was used to obtain information about the phase transition “gel to liquid crystal” (or melting) in the model lipid membranes containing GS. DSC experiments were carried out using a microcalorimeter Mettler DSC 1 (“Mettler-Toledo”). Samples (15–20 mg) were placed into a standard 40 μL aluminum crucible, sealed with a lid, and then scanned in the temperature range of 20–50 °C with a scanning rate of 1 °C/min. Several scans in “cooling–heating” mode were performed for each sample. For further processing, normalized DSC thermograms were plotted, where the original values of heat flow were normalized by the sample mass. The reproducibility of DSC profiles served as a criterion for the sample quality and stability. Based on DSC profiles, a set of thermodynamic parameters was obtained, such as melting temperature (T_m_), melting enthalpy (Δ*H_m_*), and peak half-width (ΔT_m1/2_).

### 4.3. FTIR Investigations

Fourier-transform infrared (FTIR) spectra were obtained by means of a spectrophotometer Spectrum One (“Perkin-Elmer”, Waltham, MA, USA). The samples of model lipid membranes were placed between ZnSe plates by the “crushed drop” technique and scanned in the range of 4000 to 400 cm^−1^ at room temperature. The original FTIR spectra were used after the recalculation of the values of absorbance with subtraction of the baseline (absorption of the ZnSe plates). The experimental error of wavelength values was within 1 cm^𢄣1^.

### 4.4. Statistical Analysis

The values from three to five independent preparations are presented in the figures as means ± standard deviations (SDs). Statistical significance was calculated by the Mann–Whitney test, indicating *p* < 0.05 by *.

### 4.5. Data Sharing Statement

The data supporting the findings of this study are available within the article.

## 5. Conclusions

The observed effects and proposed possibility to dynamically modify GS binding to biological membranes could be further used for optimization of the therapeutic efficacy of GS, further development of lipid-based drug-delivery nanosystems, and drug redeployment.

## Figures and Tables

**Table 1:** The cholesterol content in DPPC membranes strongly impacts GS binding, as evidenced by DSC. The thermodynamic parameters of the melting of lipid membranes containing 5 mol % GS are presented: molar membranotropic activity (a_mol_), half-width of the melting peak (ΔT_m1/2_), and shift in the melting enthalpy (ΔΔH_m1/2_). In DPPC-Chol, the content of cholesterol was 30 mol %. Typical values from one of three independent preparations are shown. Significant changes with *p* < 0.05 are indicated in bold with *.

No.	Lipid Composition	*a_mol_*, °C	Δ*T_m1/2_*, °C	ΔΔ*H_m1/2_*, kJ/mol
1	DPPC	−0.3	1.9	−12
2	DPPC-DPPG	−0.2	2.2	−14
3	DPPC-Cer	−0.2	2.1	−15
4	DPPC-Chol	−1.5 *	11.2 *	−17

**Table 2:** Qualitative changes of FTIR characteristic bands after GS (5 mol %) binding in the experimental membranes compared to the DPPC membrane. The changes in methylene, carbonyl, and phosphate groups of DPPC are shown. In the DPPC-Chol membrane, the content of cholesterol was 30 mol %. Typical changes observed in 3–5 independent preparations are shown.

	Functional Group	Methylene	Carbonyl	Phosphate
Membrane				
DPPC-DPPG		half-width ν_as_CH_2_ ↑ δCH_2_ ↑	minor changes	hydration ↓
DPPC-Cer		half-width νCH_2_ ↓ δCH_2_ ↑	minor changes	minor changes
DPPC-Chol		half-width νCH_2_ ↓ δCH_2_ ↓	hydration ↑ hypsochromic shift	hydration ↓ (compensation of Chol effect)

## Data Availability

The original contributions presented in the study are included in the article; further inquiries can be directed to the corresponding author/s.
